# Genomic analysis of the population structure of *Paenibacillus larvae* in New Zealand

**DOI:** 10.3389/fmicb.2023.1161926

**Published:** 2023-04-20

**Authors:** Barbara M. Binney, Hayley Pragert, Jonathan Foxwell, Edna Gias, Meredith L. Birrell, Bernard J. Phiri, Oliver Quinn, Michael Taylor, Hye Jeong Ha, Richard J. Hall

**Affiliations:** ^1^Animal Health Laboratory, Biosecurity New Zealand, Ministry for Primary Industries, Upper Hutt, New Zealand; ^2^Biosecurity New Zealand, Ministry for Primary Industries, Wellington, New Zealand

**Keywords:** American foulbrood (AFB), genome, sequencing, multilocus sequence typing (MLST), honey bee (*Apis mellifera* L.)

## Abstract

New Zealand is a remote country in the South Pacific Ocean. The isolation and relatively late arrival of humans into New Zealand has meant there is a recorded history of the introduction of domestic species. Honey bees (*Apis mellifera*) were introduced to New Zealand in 1839, and the disease American foulbrood was subsequently found in the 1870s. *Paenibacillus larvae*, the causative agent of American foulbrood, has been genome sequenced in other countries. We sequenced the genomes of *P. larvae* obtained from 164 New Zealand apiaries where American foulbrood was identified in symptomatic hives during visual inspection. Multi-locus sequencing typing (MLST) revealed the dominant sequence type to be ST18, with this clonal cluster accounting for 90.2% of isolates. Only two other sequence types (with variants) were identified, ST5 and ST23. ST23 was only observed in the Otago area, whereas ST5 was limited to two geographically non-contiguous areas. The sequence types are all from the enterobacterial repetitive intergenic consensus I (ERIC I) genogroup. The ST18 and ST5 from New Zealand and international *P. larvae* all clustered by sequence type. Based on core genome MLST and SNP analysis, localized regional clusters were observed within New Zealand, but some closely related genomes were also geographically dispersed, presumably due to hive movements by beekeepers.

## 1. Introduction

American foulbrood (AFB) is an important and usually fatal disease that affects honey bee colonies (*Apis mellifera*) worldwide, and caused by the bacteria *Paenibacillus larvae* (Genersch, 2010). Honey bees were introduced to New Zealand in 1839. It was not until 1907 that AFB was formally confirmed as being present in New Zealand, though reports of substantial colony losses due to “foul brood” had been made by beekeepers from the late 1870s onward (Hopkins, 1905, 1911). Systematic efforts to bring AFB under control were first introduced in New Zealand in 1906, with the introduction of regulations that required regular visual inspection of hives (Hopkins, 1911). In 1998, New Zealand legislation established a pest management plan to eliminate AFB from New Zealand (Food and Agriculture Organization, 1998). The aim of the plan is to reduce the incidence of AFB in managed honey bee colonies by 5% each year. The pest management plan requires beekeepers to register where all their beehives are located (managed *Apis mellifera* colonies) and requires a minimum of one visual inspection of every colony per year. If symptomatic AFB is observed during a visual inspection then the beekeeper is required to destroy the colony using an approved method and to notify the American Foulbrood Pest Management Agency (AFB Management Agency) administering the pest management plan^1^, within 7 days. Associated bee products and appliances may also require destruction, or sterilization. Recent years have seen the incidence of AFB in New Zealand honey bee colonies remain low. The most recent report, in 2022, of AFB annual incidence was 0.46%, that being 3,422 AFB cases notified from a total number of 736,707 colonies nationwide (King, 2022).

New Zealand has a relatively high density of hives due to plentiful floral resources, a suitable climate, and increased international demand for mānuka honey (Ausseil et al., 2018). The country has stringent border biosecurity controls that regulate the importation of bee products, where no importations of live honey bees have occurred since 1957 (Palmer-Jones, 1971), with only one instance of honey bee germplasm being imported in 2004 (Yanke, 2005).

International studies of genome data from *Paenibacillus larvae* have made good progress within the last 10 years (Djukic et al., 2014; Perez de la Rosa et al., 2015; Agren et al., 2017; Papic et al., 2021; Zugelj et al., 2021). Such studies provide multi-locus sequence genotypes (MLST) that can be used to track clusters of disease, and also provide higher-resolution single nucleotide polymorphism (SNP) data and other information on other elements such as bacteriophage or virulence genes. Given the increase in genomic information available for *P. larvae* in other regions of the world, and the unique management and production aspects of the New Zealand honey bee population, we sought to characterize the genetic structure of *P. larvae* circulating in New Zealand.

## 2. Materials and methods

### 2.1. Sample collection

Twenty one apiculture inspectors, accredited under the New Zealand biosecurity legislation, collected samples for this study. The inspectors were routinely conducting visual hive inspections for symptomatic American Foulbrood (AFB). When an inspector encountered symptomatic AFB in a colony, they took a sample of an infected larva or pupa. Only a single sample was taken at each apiary. Samples were collected between November 2019 and January 2022. The date of sampling and the geographic subregion of New Zealand, according to the boundaries defined by Crosby et al. (1998), were recorded (Supplementary Figure 1). Samples were obtained from 22 subregions of New Zealand, namely, Auckland, Buller, Central Otago, Coromandel, Dunedin, Gisborne, Hawkes Bay, Marlborough, Mid Canterbury, Nelson, North Canterbury, Northland, Otago Lakes, South Canterbury, Southland, Taranaki, Taupo, Waikato, Wairarapa, Whanganui, Wellington, and Westland.

### 2.2. Microbiological culture of *Paenibacillus larvae*

Collected samples were emulsified in 1 ml Dulbecco’s Phosphate Buffered Saline (DPBS) (Gibco, Life Technologies, Paisley, UK) and heat-shocked at 80°C for 10 min in order to kill vegetative forms of other microorganisms, including other spore formers (World Organisation of Animal Health, 2018). Loopfuls of these heat-shocked samples were then inoculated onto non-selective Columbia Sheep Blood Agar (BA), selective Sheep Blood Agar with 3 μg/mL Nalidixic acid (SBANA3) and selective Sheep Blood Agar with 18 μg.mL^–1^ Nalidixic acid (SBANA18) (Fort Richard Laboratories, Auckland, New Zealand) in parallel. All media was incubated at 37°C, 5% CO_2_, for minimum 48 h. ATC strain 9545 *Paenibacillus larvae* was inoculated on each media as positive control.

Colonies were first inspected macroscopically and screened based on colony morphology (World Organisation of Animal Health, 2018), in comparison to that of the positive control. Any possible putative colonies were analyzed using a catalase test (Catalase dropper; Becton, Dickinson & Co., Franklin Lakes, NJ, USA). Fresh sub-cultures of catalase-negative isolates were further characterized by MALDI-TOF Mass Spectrometry (MALDI Biotyper^®^; Bruker, Billerica, MA, USA). Confirmed *P. larvae* isolates were preserved as a reference collection (−80°C, CRYOBANK^®^ beads; Mast Group, Liverpool, UK) and further processed for DNA extraction, for genomic sequencing.

### 2.3. DNA extraction

Bacteria cells were washed with 1 ml of DPBS (Gibco, Life Technologies, Paisley, UK) and pelleted by centrifugation at 20,000 × *g* for 1 min. DNA was extracted from the bacterial pellet using QIAamp^®^ DNA Mini Kit (Qiagen, Hillden, Germany) following the manufacturer’s instructions for extraction from Gram-positive bacteria. Briefly, the pellet was resuspended in 180 μL of 20 mg.mL^–1^ lysozyme solution and incubated at 37°C for 1 h. Lysis was performed by incubating the suspension in 20 μL proteinase K and 200 μL Buffer AL at 56°C for 2 h, or overnight, followed by 95°C for 15 min. After the addition of 200 μL ethanol, the DNA bound on the silica membrane was washed twice with Buffer AW1 and AW2. The DNA was eluted in 50 μL of 10 mM Tris–HCl, pH 8.0 after 5 min incubation at room temperature and stored at -20°C if not used immediately. The concentration and purity of DNA was measured using Qubit™ HS DNA assay (Life Technologies, Eugene, OR, USA) and Nanodrop (Thermo Fisher Scientific, Wilmington, DE, USA), respectively.

### 2.4. DNA library preparation

DNA of between 1 and 500 ng in a final volume of 30 μL was used as input for library preparation using Illumina DNA Prep kit (Illumina, San Diego, CA, USA). Briefly DNA was tagmented using bead-linked transposomes (BLT). After cleaning up with tagment wash buffer the adaptor-tagged DNA was indexed and amplified in Enhanced PCR Mix and index adapters provided in the Nextera DNA CD Indexes kit (Illumina, San Diego, CA, USA). The number of PCR cycles used varied according to the amount of input DNA, as recommended by the manufacturer. Following the clean-up with sample purification beads, the concentration and quality of the libraries were assessed using the Qubit HS DNA assay and HS D5000 ScreenTape assay (Agilent Technologies, Santa Clara, CA, USA) on a TapeStation System (Agilent Technologies, Santa Clara, CA, USA), respectively.

### 2.5. Genome sequencing and analysis

Whole genome sequencing of the *P. larvae* isolates was carried out using MiSeq (Illumina, San Diego, CA, USA). DNA libraries were pooled to an equimolar concentration of 2 nM or 4 nM. Pooled libraries were denatured with 0.2 M NaOH and diluted to 20 pM in HT1 buffer. The libraries were further diluted to a loading concentration between 10 and 18 pM followed by spiking with 1% of 12.5 pM PhiX. The libraries were sequenced using MiSeq Reagent v2, 500 cycles chemistry (Illumina, San Diego, CA, USA).

Short reads were checked using FastQC v0.11.9 (Andrews, 2010) for quality. Trimmomatic v0.39 removed Illumina adapters as well as nucleotides at both ends with Phred score < 15 (Bolger et al., 2014). Using a sliding window, 4 bp reads with an average quality < 15 were clipped and reads shorter than 100 bp were removed using Trimmomatic v0.39 (Bolger et al., 2014). The output files were combined into a single report by MultiQC v1.8 (Ewels et al., 2016) and reviewed. The reads were assembled by the Shovill v0.7.17r1188 pipeline (Seeman, 2017), using SPAdes v3.14.1 (Bankevich et al., 2012) for the *de novo* assembly. Standard settings were used, including a minimum contig size of 100 bp and minimum coverage of 10. QUAST (v5.0.2) (Gurevich et al., 2013), checkM (Parks et al., 2015), and seqkit (Shen et al., 2016) were used to check the draft genome quality and generate their statistics. Mean genome coverage was calculated by Qualimap 2 (Okonechnikov et al., 2016), after the trimmed paired reads were mapped to the draft genomes by Bowtie2 (Langmead and Salzberg, 2012) and SAMtools (Li et al., 2009). Annotation was performed using Prokka (Seemann, 2014).

### 2.6. *In silico* 7-gene multi-locus sequence typing

Each isolate was assigned a multi-locus sequence type (MLST) according to the 7-gene scheme designated for *P. larvae* in the PubMLST database (Jolley et al., 2018) (downloaded 10 December 2021). The 7-genes were extracted *in silico* from the genome of each isolate and mapped to known alleles using SRST2 (Inouye et al., 2014). New alleles were checked in Geneious 2021.1.1^2^ and submitted to PubMLST.

### 2.7. Core genome analysis

Roary v3.13.0 (Targe, 2011; Page et al., 2015) was used to define a core genome for the 163 New Zealand isolates with a 95% identity value cut off. The core genome data generated by Roary was converted into a cgMLST (core genome MLST) format by roProfile (Mendes, 2016). GrapeTree created a minimum spanning tree (MST) from the cgMLST (Zhou et al., 2018).

### 2.8. Phylogenetic analysis

We used ModelTest-NG to determine the best-fit evolutionary model by AIC and BIC values as GTR + I + G4 (Darriba et al., 2020). A maximum likelihood tree was made using this model in RaxML v1.0.1 (Kozlov et al., 2019) on the core genome alignment from Panaroo (Tonkin-Hill et al., 2020) using 163 NZ genomes and 20 from Papic et al. (2021).

### 2.9. Population structure and Geospatial analysis

The genetic population structure was inferred from a core genome alignment using a Bayesian hierarchical clustering approach in RhierBAPS v1.1.3 (Tonkin-Hill et al., 2018) implemented in R v.4.1.2.

## 3. Results

### 3.1. Regional distribution of *Paenibacillus larvae* sequence types in New Zealand

**FIGURE 1:**
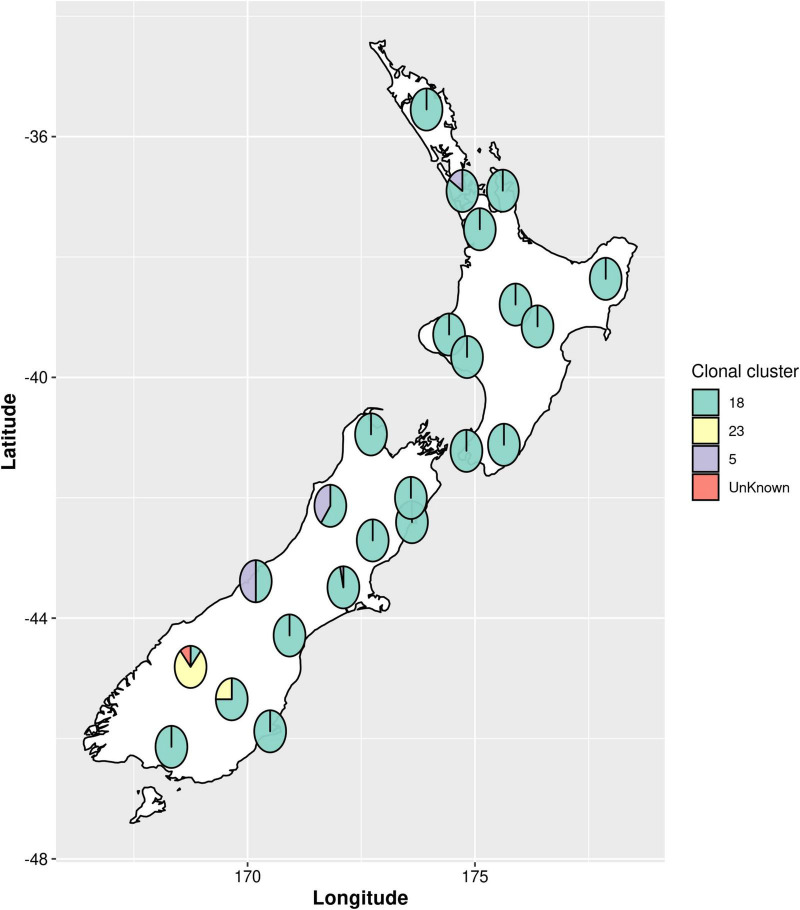
Geographic distribution of *Paenibacillus larvae* multi-locus sequence types (MLST) across New Zealand. Pie charts show the proportions of sequence types present in each region. The sequence types are grouped as clonal clusters. Each clonal cluster consists of the main sequence type (ST) and the single nucleotide polymorphism (SNP) variants of the main sequence type. An untypable genome, referred to in the figure as “unknown”, shared six loci with ST23 but the seventh loci was not found.

All samples collected were culture-positive for *Paenibacillus larvae.* Bacterial colony morphology was, in all cases, that of the typical enterobacterial repetitive intergenic consensus I (ERIC I) strain ATCC 9545. Whole genome sequence was derived for all *n* = 164 *P. larvae* isolates. One isolate was excluded from all analyses except for the multi-locus sequence typing (MLST) genotyping due to poor quality of the resultant sequence data. MLST analysis revealed three main sequence type clusters (Figure 1), ST5 (*n* = 7), ST 18 (*n* = 147) and ST23 (*n* = 9), and an untypable sequence (*n* = 1). The untypable genome shared six loci with ST23, but the seventh loci was not found. Each of these sequence types are within the ERIC I genogroup (Morrissey, 2015; Morrissey et al., 2015; Beims et al., 2020; Papic et al., 2021). ST18 was the most common sequence type, accounting for 90.2% of the isolates. ST5 and ST23 were less common, with ST23 being geographically restricted to the Otago Lakes region, in the South Island (Table 1). Numerous SNP variants were observed, with ST18 having eight 1-SNP variants and one 2-SNP variant, ST5 had a single 1-SNP variant and ST23 having two 1-SNP variants (Figure 1 and Table 1). These will represent new sequence types, but they remain part of their clonal cluster (MLST submitted to PubMLST).

**TABLE 1:** Regional distribution of multilocus sequence types (MLST) of *Paenibacillus larvae* in New Zealand.

MLST	Number of isolates with ST	Number of regions with ST	Name of regions with ST
18	135	22	All 22 subregions
18 _clpC	3	2	Kaikoura, Mid Canterbury
18 _glpT1	1	1	Marlborough
18 _glpT2	2	2	Whanganui, Waikato
18 _glpT3	1	1	Buller
18 _Natrans1	1	1	Mid Canterbury
18 _Natrans2	1	1	Waikato
18 _Natrans3	1	1	Southland
18 _rpoB	1	1	Nelson
18 _rpoB _NaTrans	1	1	Nelson
23	8	2	Otago Lakes, Central Otago
23 _rpoB	1	1	Otago Lakes
5	6	4	Mid Canterbury, Auckland, Buller, Westland
5 _sigF	1	1	Auckland
Untypable	1	1	Otago Lakes

### 3.2. Core genome multilocus sequence type (cgMLST)

**FIGURE 2:**
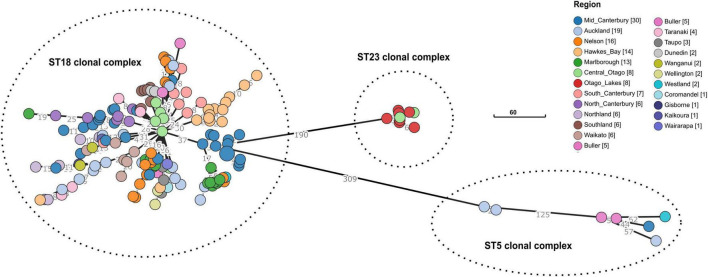
Minimum-spanning of the core genome multilocus sequence types (cgMLST) for *n* = 163 *Paenibacillus larvae* in New Zealand, by region. Regions are color coded, with the tally of isolates shown in square brackets. The dashed lines delimit three clusters, each representing one of the 7-gene MLST we found in this study (viz., ST5, ST18, and ST23). The total number of allele differences between each node are shown on the branch lines. Scale bar shows 60 allele differences.

The cgMLST for the 163 New Zealand genomes in this dataset was found to be 2,891 loci. The cgMLST analysis showed that the isolates clustered according to their sequence type: ST5, ST18, ST23 (Figure 2). There are indications of geographical clustering within the dominant ST18 group, as appears evident in Hawkes Bay, South Canterbury, and Southland. The Otago region (Central Otago/Otago Lakes) was distinct from the rest of New Zealand because it contained an isolated population of ST23. However, the Otago region also contained the common ST18. ST5 whilst uncommon in New Zealand but highly dispersed, being found in both the North and South Islands.

### 3.3. Population structure

**FIGURE 3:**
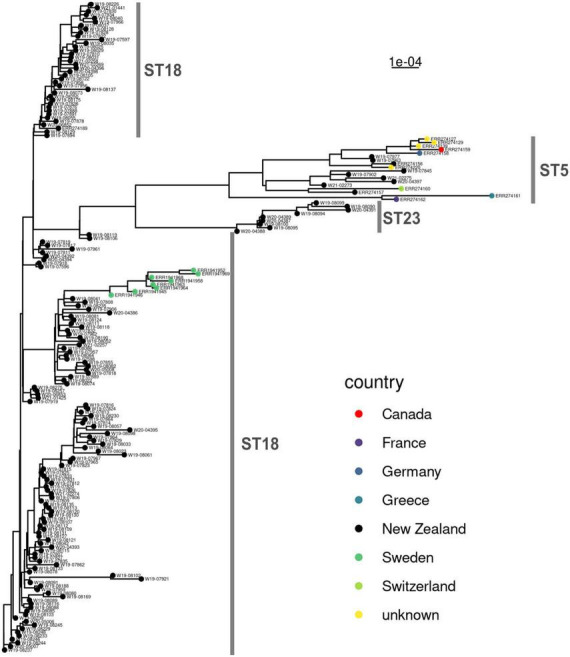
Maximum-likelihood phylogenetic analysis comparing *n* = 163 New Zealand and *n* = 20 international genomes of *Paenibacillus larvae*. The scale bar shows the genetic distance. The multilocus sequence types are delineated in gray, alongside the phylogenetic tree.

International WGS (whole genome sequence) data from isolates that are ST18 and ST5 (Papic et al., 2021) were compared to the New Zealand genomes in this study. No WGS data could be found for ST23 (apart from the ST23 identified in our study). A maximum-likelihood phylogenetic tree of the core genomes (Figure 3) produced a result consistent with the cgMLST analysis (Figure 2). The New Zealand and international genomes consistently grouped by sequence type. There were some localized geographic clusters also evident within New Zealand.

**FIGURE 4:**
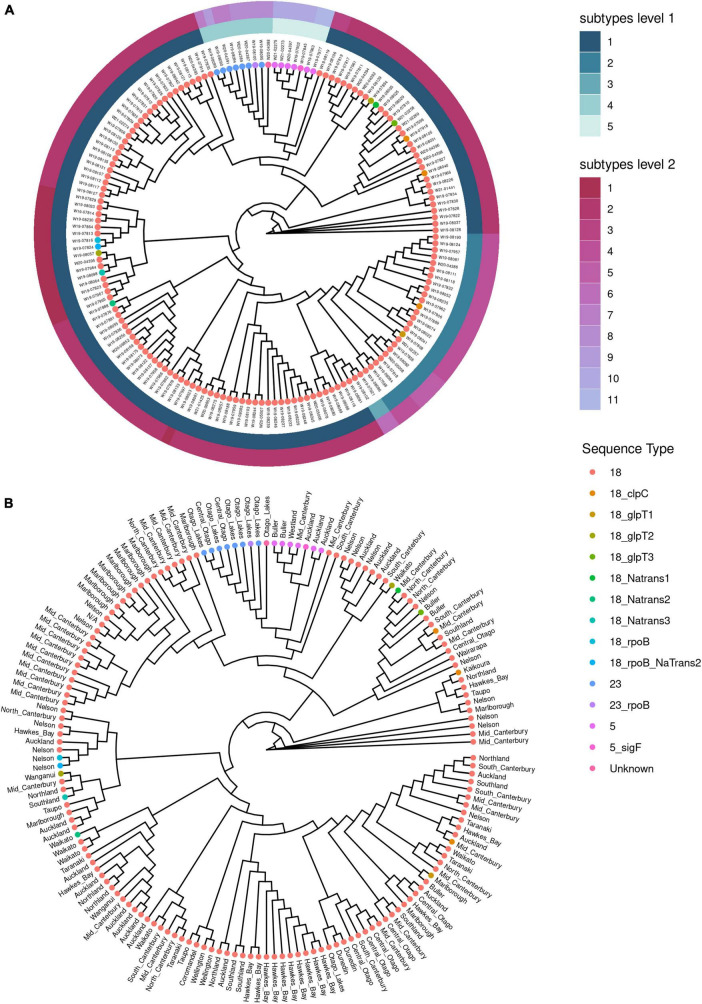
Population structure analysis using hierarchical clustering of core genomes for *Paenibacillus larvae* from New Zealand. Clustering was based on analysis of single nucleotide polymorphisms. **(A)** Cladogram shown with subtypes assigned by hierarchical clustering analysis using RhierBAPS. Sequence types (ST) are coded by color for each isolate, and annotated with the unique genome identifier. **(B)** Cladogram by hierarchical clustering analysis using RhierBAPS without subtype assignment, and with the region of New Zealand annotated for each isolate.

Further investigation of the population structure using hierarchical clustering showed that ST18, ST5 and ST23 could be divided into subtypes (Figure 4A). There are five subtypes at level 1, with ST18 being the only ST with subdivisions at level 1. At a more sensitive subtype detection, level 2, the ST18 group resolved into further subtypes, as did ST5 and ST23. This shows a population structure within each of the sequence type clusters, as was also observed in the cgMLST analysis (Figure 2). Evidence for geographic clustering was also observed in the hierarchical clustering method (Figure 4B).

## 4. Discussion

We sequenced whole genomes from 164 *P. larvae* collected from throughout New Zealand between 2020 and 2022, and found three sequence types (ST18, ST5, and ST23) with associated SNP variants. Clonal expansion of the three sequence types over time has generated these SNP variants. All three sequence types have been found in other countries and belong to the ERIC I genogroup (Papic et al., 2021). We sequenced 37.9% of all AFB-positive apiaries in New Zealand that were inspected by the AFB management agency during the study period, and therefore provide a good representation of the genotypes that were circulating in New Zealand at the time.

ERIC typing is a conventional PCR-based method for broadly categorizing *P. larvae* strains prior to MLST (Papic et al., 2021). ERIC I has higher virulence at the colony-level, compared to ERIC II which displays a higher virulence at the individual larval level (Rauch et al., 2009). ERIC I is the most common AFB genogroup found internationally, but some countries have reported ERIC II as the most common genogroup (Zugelj et al., 2021). If ERIC II is present in New Zealand, then it is circulating at a lower level than could be detected in our study. Only two other studies have examined ERIC types in New Zealand (Graham, 2014; Morrissey et al., 2015) and both have reported the presence of ERIC II. One of these studies used New Zealand honey samples (*n* = 16) and found MLST genotypes ST1, ST3, ST5, ST13, ST18, and ST10 (Morrissey et al., 2015); the ST10 being from the ERIC II genogroup (Papic et al., 2021). It is surprising that Morrissey et al. (2015) found six different sequence types in only 16 honey samples, whereas we found only three sequence types (with SNP variants) in 164 apiaries. A possible explanation could be due to the provenance of the New Zealand honey samples tested by Morrissey et al. (2015)—given what is known about blending and/or the misrepresentation of New Zealand honeys which can occur in the international marketplace (Burns et al., 2018; McDonald et al., 2018). The other study, unpublished, by Graham (Graham, 2014) used rep-PCR with the MBO REP1 primers to infer ERIC type but did not use the ERIC primers (Biova et al., 2021). This leaves three hypotheses that could explain why ERIC II was not detected in our study: (a) ERIC II was never present in New Zealand, (b) ERIC II has become extinct due to the national AFB eradication program, (c) ERIC II strains of AFB do exist in New Zealand but are rare, and therefore our study did not detect them due to chance.

In our study, ST23 was only found in the Central Otago and Otago Lakes districts of the South Island. It has only ever been previously recorded in Scotland (Morrissey, 2015). It should be noted that ST18, and SNP-variants thereof, were also present in Central Otago and the Otago Lakes districts. It is unclear why ST23 is limited to this geographic area, it may be present in other areas of New Zealand that we had not sampled. It is possible that it has been eliminated from other areas through the AFB eradication program, by pure chance.

Sequence type 5 (ST5) was an uncommon sequence type in our study, with a discontiguous geographic distribution. It was found in the Auckland region of the North Island, and then some 600 km distant in the West Coast and mid-Canterbury regions of the South Island. Given that it is uncommon in our study, we may not have revealed the actual distribution of ST5, which could be present in other regions. We suggest that hive movements account for the disjointed geographic distribution pattern. Hives are moved by beekeepers across the Southern Alps (an extensive mountain range creating a significant geographic barrier in the South Island) between the West Coast and Canterbury, to pursue rata, kamahi, mānuka and honeydew flows (*pers. comm.* Marco Gonzalez). Wider hive movements are also known to occur, including movement across Cook Strait which separates the North and South Islands. We suggest this accounts for the wide distribution of ST18, and the ST18 SNP-variants. We cannot be precise about where transmission events have occurred, even though it appears there is some evidence for regional geographic clustering in the cgMLST data. Our study shows that there is enough variation in the MLST and the wider genome to be informative for outbreak investigations of American foulbrood in New Zealand, as has been shown for other countries (Agren et al., 2017; Papic et al., 2021; Zugelj et al., 2021). In summary, despite New Zealand’s isolation the ST5 and ST18 within the country still cluster with international ST5 and ST18.

New Zealand management of American foulbrood is unique as it aims to eliminate the disease (Food and Agriculture Organization, 1998). All beekeepers are required by law to destroy infected hives on discovery. Antibiotic use is prohibited in New Zealand beehives (Hall et al., 2021). We consider the AFB elimination strategy over the past 24 years has impacted the population structure of *P. larvae*. It is likely that the natural evolution and spread of *P. larvae* strains has been disrupted. While some strains have survived others could have become extinct. These factors could partly account for finding only three sequence types, with MLST SNP-variants. The MLST SNP-variants follow a pattern of clonal expansion from the three original sequence types (ST18, ST5, and ST23) that could be due to geographic isolation and tight border biosecurity.

When considering our analysis, we suggest that the most-common reason for dispersal of AFB in New Zealand, at a national-level, is the movement of hives by beekeepers. There is also clearly a local effect where genotypes cluster within regions, which may be brought about by natural behaviors of honey bees such as robbing (and to extent, drift), which are usually thought to be greatest within a 1 kilometer radius from affected hives (Goodwin et al., 1994; Lindström et al., 2008). To this effect, it is known that once AFB arrives in an area, early inspections and detection of the disease may help to limit local spread (Datta et al., 2013).

Our study is the first comprehensive genomics description of *P. larvae* in New Zealand. The data in this study also makes a substantial contribution to the international dataset of *P. larvae* whole genomes. We suggest that the wide geographic distribution of ST18 within New Zealand is due to the large numbers of hive movements around the country. We propose that a network analysis of hive movements alongside genomic tracing of disease spread, would assist in efforts to eliminate American foulbrood from New Zealand. This would also hold benefits not just for AFB control, but potentially also for responding to incursions of exotic diseases or pests. Electronic tracing of hive movements is already used by large beekeeping operations, and hive movements are a known major dispersal mechanism for many honey bee pests and diseases.

## Data availability statement

The datasets presented in this study can be found in online repositories. This data can be found here: https://www.ncbi.nlm.nih.gov/bioproject/PRJNA949734.

## Author contributions

HP, RH, HH, and MT developed and designed the study. HP, RH, and BP developed the sampling plan. JF, EG, MB, and OQ undertook the laboratory testing and analysis. BB and RH undertook the bioinformatics analysis and wrote the manuscript. All authors contributed to the analysis and interpretation of the results from the study, revised the manuscript, and approved the submitted version.

## Footnotes

1
https://afb.org.nz/

2
https://www.geneious.com/
