# Supplementary figures and images for: Genomic analysis of the population structure of Paenibacillus larvae in New Zealand
Source: Front Microbiol. 2023 Apr 20;14:1161926. doi: 10.3389/fmicb.2023.1161926 (PMC10157257; doi:10.3389/fmicb.2023.1161926)

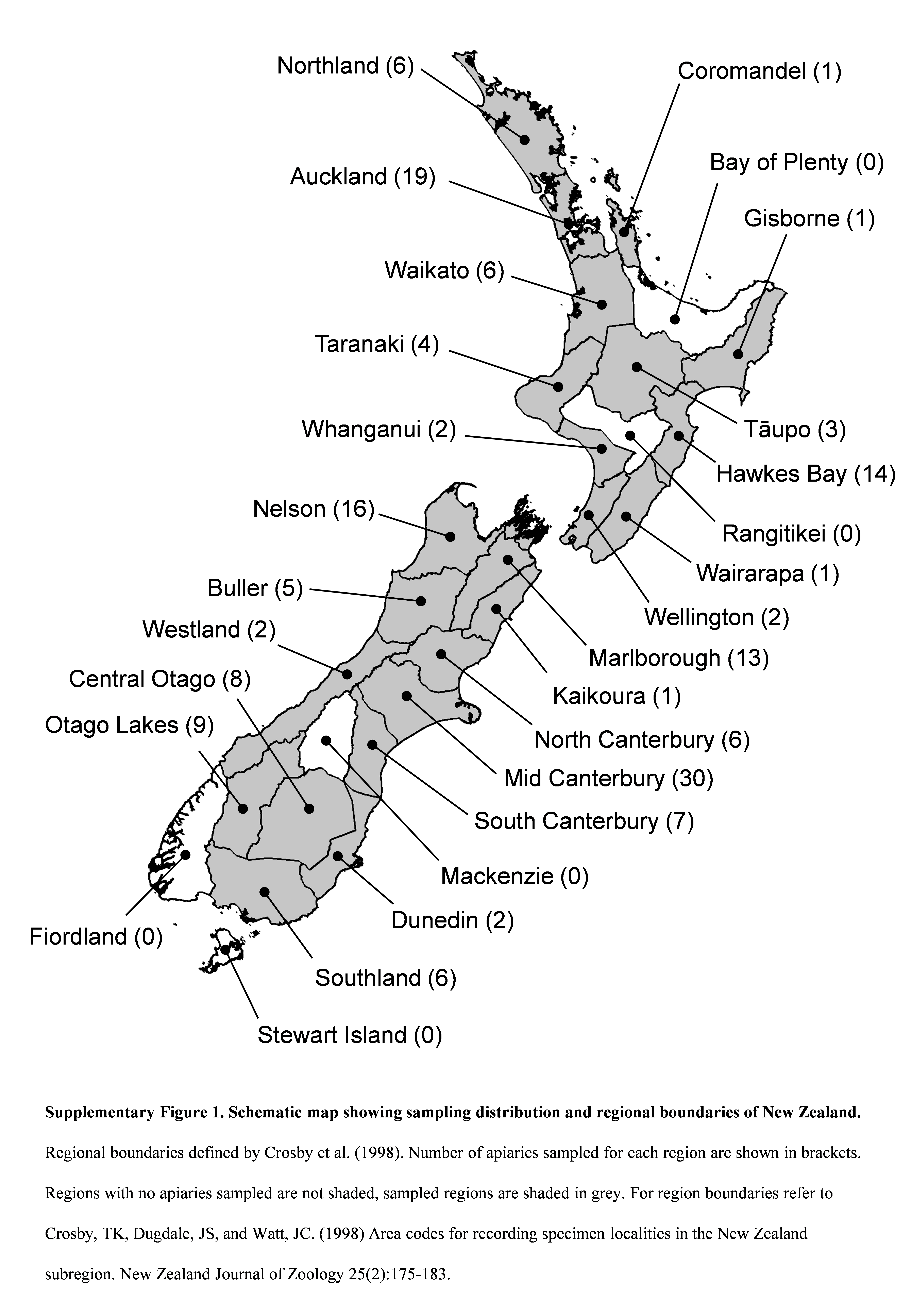

Supplement: Supplementary file 1 [file Image_1.TIF]
